# Effectiveness of Negative Pressure Wound Therapy and Advanced Interventions in Preventing Surgical Site Infections: A Systematic Review

**DOI:** 10.7759/cureus.95906

**Published:** 2025-11-01

**Authors:** Muaz Hassan, Mohamed Eltayeb Abdelrahman Naiem, Moustafa U Abdelradi, Rasha Omer Babiker Mohamed, Mohamed Ibrahim Osman Hamd, Rouida Aboagla

**Affiliations:** 1 General Surgery, Alnao Teaching Hospital, Khartoum, SDN; 2 General Surgery, Faculty of Medicine, University of Khartoum, Khartoum, SDN; 3 Vascular Surgery, Aberdeen Royal Infirmary, Grampian NHS Trust, Aberdeen, GBR; 4 Pediatric Intensive Care Unit, Dr. Sulaiman Al Habib Medical Group, Riyadh, SAU; 5 Trauma and Orthopedics, Nobles Hospital, Douglas, IMN; 6 General Surgery, Almoweh General Hospital, Taif, SAU; 7 Pediatric Surgery, Egypt Children’s Hospital, Cairo, EGY

**Keywords:** advanced wound dressings, incisional npwt, negative pressure wound therapy, randomized controlled trial, surgical site infection, systematic review

## Abstract

Surgical site infections (SSIs) are a major cause of postoperative morbidity and increased healthcare costs. Negative pressure wound therapy (NPWT) and other advanced wound dressings have been widely adopted to mitigate this risk; however, evidence of their effectiveness across diverse surgical settings remains inconsistent. This systematic review aims to evaluate the effectiveness of NPWT and advanced interventions in preventing SSIs compared to conventional dressings or standard care. A systematic search was conducted across five electronic databases and registers (PubMed, Excerpta Medica database (Embase), Scopus, Web of Science, ClinicalTrials.gov) for randomized controlled trials (RCTs) published up to 2025. Studies comparing NPWT or advanced dressings to conventional care for SSI prevention in any surgical population were included. Study selection, data extraction, and risk of bias assessment using the Cochrane Risk of Bias 2 (RoB 2) tool were performed by independent reviewers. A narrative synthesis was conducted due to clinical heterogeneity. Eight RCTs were included, encompassing procedures such as emergency laparotomy, vascular surgery, cesarean section, and orthopedic trauma. The results demonstrated that the effectiveness of advanced interventions is highly context-dependent. NPWT significantly reduced SSIs in open inguinal vascular surgery, and a dialkylcarbamoyl chloride (DACC)-impregnated dressing was effective following cesarean section. In contrast, the largest RCT found no benefit of incisional NPWT after emergency laparotomy. Evidence in orthopedic trauma was mixed. The majority of included trials were assessed as having a low risk of bias. The effectiveness of NPWT and advanced wound interventions in preventing SSIs is not universal but is contingent on the specific surgical context. These technologies should be applied selectively, targeting patient populations and procedures where a clear benefit has been demonstrated, rather than being used routinely. Future research should focus on identifying predictive factors to guide the cost-effective, personalized application of these therapies.

## Introduction and background

Surgical site infections (SSIs) remain one of the most significant complications following surgical procedures, contributing to increased morbidity, prolonged hospital stays, and substantial healthcare costs worldwide [1]. Despite advances in surgical techniques, aseptic practices, and perioperative care, SSIs continue to affect approximately 2%-5% of patients undergoing clean or clean-contaminated surgeries, with higher rates observed in complex or high-risk procedures [2]. The prevention of SSIs is therefore a critical component of perioperative management and patient safety initiatives.

Negative pressure wound therapy (NPWT) has emerged as a promising strategy for reducing the incidence of SSIs, particularly in patients undergoing high-risk or complex surgical interventions [3]. NPWT involves the application of subatmospheric pressure to the wound bed via a sealed dressing, promoting fluid drainage, enhancing perfusion, and stimulating tissue granulation [4]. Over the past decade, NPWT has been increasingly utilized as either a prophylactic measure in closed surgical incisions or as a therapeutic intervention for managing postoperative wounds, with growing evidence suggesting its potential to decrease infection rates and improve wound healing outcomes [5].

In addition to NPWT, various advanced wound care interventions, including antimicrobial dressings, biologic therapies, and novel barrier technologies, have been developed to mitigate SSI risk [6]. These interventions target different aspects of wound healing, such as microbial colonization, local inflammation, and tissue regeneration, aiming to enhance postoperative recovery and reduce the burden of infection [7]. However, the relative effectiveness of NPWT compared with these advanced interventions and standard care remains a topic of ongoing investigation.

Given the expanding body of literature and the clinical implications of SSIs, a systematic synthesis of recent randomized controlled trials (RCTs) and high-quality studies is warranted. This systematic review aims to evaluate the effectiveness of NPWT and other advanced interventions in preventing SSIs, comparing their outcomes with conventional dressings or standard care, and providing evidence-based recommendations for optimizing postoperative wound management.

## Review

Methodology

Eligibility Criteria

This systematic review focused on RCTs evaluating the effectiveness of NPWT and advanced interventions in preventing SSIs across various surgical populations. RCTs were specifically chosen due to their ability to minimize confounding factors and provide the highest level of evidence regarding intervention efficacy. Trials involving both adult and pediatric patients undergoing any type of surgical procedure were included, while observational studies, case reports, and reviews were excluded to maintain methodological rigor.

Information Sources and Search Strategy

A comprehensive literature search was conducted in ClinicalTrials.gov, PubMed, the Excerpta Medica database (Embase), Scopus, and Web of Science to identify relevant studies. Additional records were identified through citation tracking of eligible studies to ensure no significant publications were overlooked. Search strategies combined Medical Subject Headings (MeSH) and free-text terms related to “negative pressure wound therapy,” “advanced wound care,” “surgical site infections,” and “randomized controlled trials.” The search strategy was tailored to each database to maximize sensitivity and ensure comprehensive coverage of the literature. The detailed search strategies for the databases are presented in Appendix A. 

Study Selection

All retrieved records were imported into EndNote X9 (Clarivate, Philadelphia, PA), and duplicate entries were removed. Two independent reviewers screened titles and abstracts for relevance, followed by a full-text review of potentially eligible studies. Discrepancies were resolved through discussion or consultation with a third reviewer to achieve consensus. This rigorous selection process ensured that only high-quality RCTs addressing the primary objective of SSI prevention were included in the final analysis.

Data Extraction

Data extraction was performed independently by two reviewers using a standardized extraction form. Extracted information included study characteristics (country, sample size, surgical type, intervention, comparator details, and follow-up duration) and key outcomes (SSI rates, wound healing time, length of hospital stay, and other relevant clinical endpoints). Crucially, the specific criteria and method used for diagnosing SSIs (e.g., Centers for Disease Control and Prevention (CDC) criteria, clinical assessment, culture confirmation) in each study were also extracted. The use of standardized forms and double extraction minimized errors and ensured consistency across included studies.

Risk of Bias Assessment

The risk of bias in included RCTs was assessed using the Cochrane Risk of Bias 2 (RoB 2) tool [8]. This assessment was performed independently by two reviewers. This instrument evaluates bias across domains such as randomization, deviations from intended interventions, missing outcome data, measurement of outcomes, and selective reporting. Each domain was rated as low, some concerns, or high risk of bias. Any discrepancies between reviewers were resolved through discussion or by consulting a third reviewer. This systematic assessment provided a transparent appraisal of study quality and reliability of reported outcomes.

Data Synthesis

Although data from multiple RCTs were available, the heterogeneity in patient populations, surgical types, intervention protocols, outcome definitions, and follow-up durations precluded quantitative synthesis through meta-analysis. Differences in SSI assessment criteria, timing of outcome evaluation, and variations in NPWT and advanced dressing modalities meant that pooled effect estimates could be misleading. Therefore, a narrative synthesis was conducted to summarize and compare the findings of individual studies, providing a qualitative overview of intervention effectiveness while highlighting trends and gaps in the current literature.

Results

Study Selection Process

**Figure 1:**
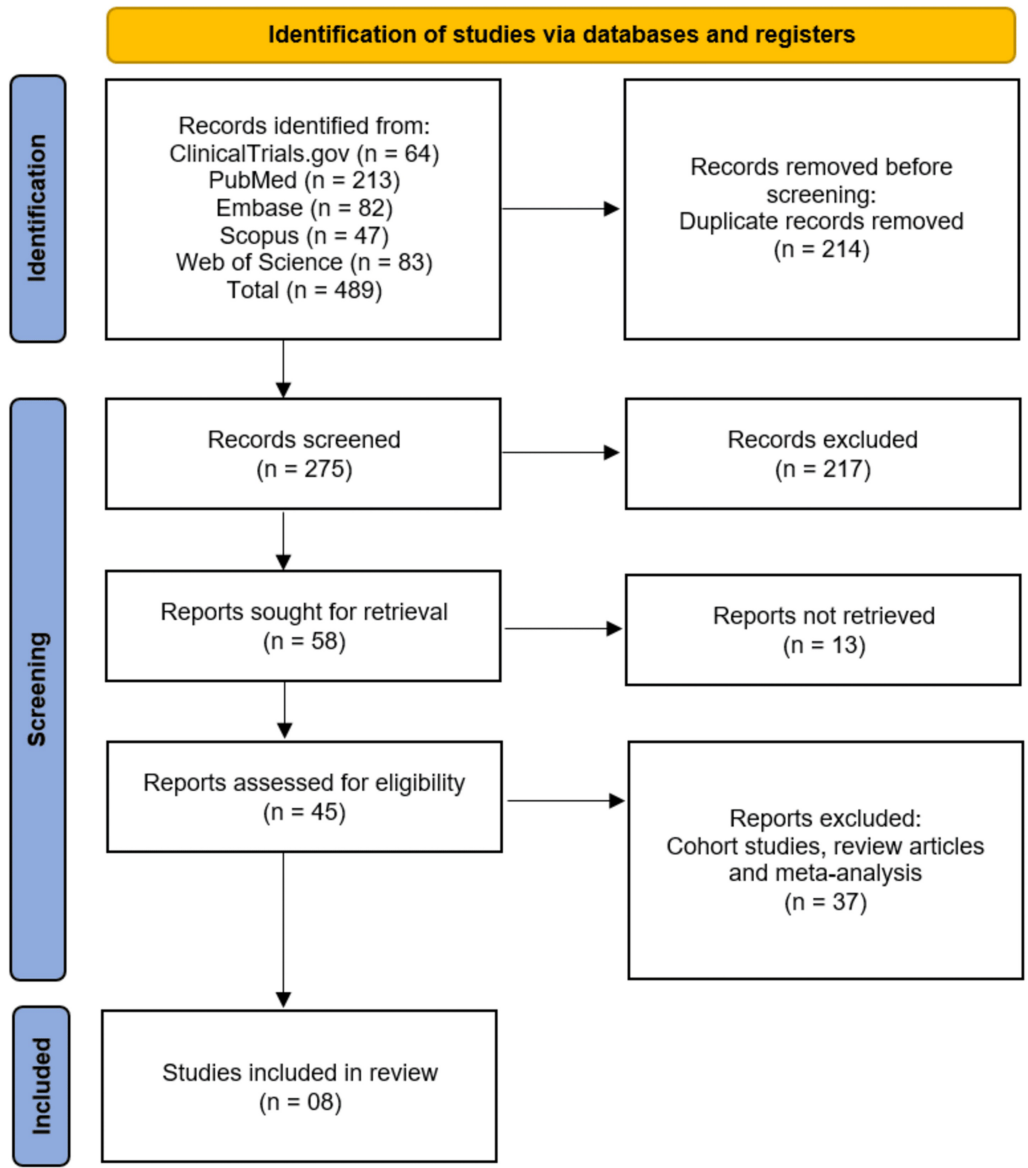
Flowchart of the study identification, screening, and inclusion process following the PRISMA 2020 statement PRISMA: Preferred Reporting Items for Systematic Reviews and Meta-Analyses

A systematic search was conducted across five electronic databases and registers (PubMed, Embase, Scopus, Web of Science, and ClinicalTrials.gov) for RCTs published from database inception until October 2025. This search initially identified 489 records. After the removal of 214 duplicate records, a total of 275 unique citations were screened based on their titles and abstracts. This screening process led to the exclusion of 217 records that did not meet the inclusion criteria. The full texts of the remaining 58 reports were sought for retrieval, of which 13 could not be acquired. Consequently, 45 full-text articles were assessed for eligibility. Upon detailed evaluation, 37 reports were excluded as they were cohort studies, review articles, or meta-analyses, leaving a final total of eight RCTs for inclusion in this systematic review (Figure 1) [9-16].

The systematic review included eight RCTs [9-16] that evaluated the effectiveness of NPWT and other advanced wound dressings in preventing SSI. The key characteristics of these studies are summarized in Table 1. The trials were conducted across a range of countries, including the United Kingdom, Australia, Sweden, Poland, the United States, Kenya, and Iran, reflecting a diverse global perspective [9-16]. Sample sizes varied considerably, from 32 participants in a study on lower extremity reconstruction [13] to 840 in a large multi-center trial on emergency laparotomy [9].

**Table 1:** Characteristics of included randomized controlled trials CDC: Centers for Disease Control and Prevention; CS: cesarean section; DACC: dialkylcarbamoyl chloride; EVAR: endovascular aortic repair; iNPWT: incisional negative pressure wound therapy; n: sample size; NAA: nonadherent absorbable; NPWT: negative pressure wound therapy; NR: not reported; PTG: paraffin-tulle gras; SSD: standard surgical dressing; SSI: surgical site infection; UK: United Kingdom; USA: United States of America; and VascuQol-6: Vascular Quality of Life Questionnaire-6

Author (Year)	Country / Region	Sample Size (n)	Surgical Type / Population	Intervention (NPWT/Other Advanced Therapy)	Comparator (Conventional Dressing/Standard Care)	Follow-Up Duration	Primary Outcome(s)
Atherton et al., [9] (2025)	United Kingdom (22 hospitals) and Australia (12 hospitals)	840 randomized (788 analyzed: 394 iNPWT vs 394 control)	Adult patients undergoing emergency laparotomy with primary skin closure	iNPWT using a specialized dressing to create negative pressure over the closed wound	Surgeon’s choice of wound dressing (standard/conventional care)	30 days post procedure	SSI rate up to 30 days post procedure (CDC criteria)
Svensson-Björk et al., [10] (2022)	Sweden	377 incisions (336 bilateral, 41 unilateral)	Patients undergoing elective EVAR with closed inguinal incisions	NPWT applied to closed incisions	Standard dressing (conventional postoperative wound dressing)	90 days postoperatively (with additional monitoring up to one year)	SSI incidence
Svensson-Björk et al., [11] (2021)	Sweden and the UK	119 (NPWT: 59; Control: 60)	Patients undergoing open inguinal vascular surgery	NPWT applied to closed surgical incisions	Standard dressings (conventional postoperative wound care)	90 days postoperative	Incidence of SSI and cost-effectiveness (vascular procedure-related costs, quality of life via VascuQol-6)
Stanirowski et al., [12] (2016)	Poland (Mazovian Bródno Hospital)	543	Women undergoing elective or emergency CS	DACC impregnated dressing	SSD	Postoperative SSI monitoring	SSI rate, cost-effectiveness
Rezzadeh et al., [13] (2015)	USA	32	Patients with Gustilo class IIIB or IIIC open tibial fractures undergoing lower extremity reconstruction	NPWT	Wet-to-dry dressing changes	NR	Flap complications, infection, nonunion, number of surgical procedures, and length of hospitalization
Ondari et al., [14] (2016)	Kenya (Kenyatta National Hospital)	77 (40 in the 24-hour group, 37 in the five-day group)	Gustilo II open tibia fractures, patients aged 18–80 years	Prophylactic antibiotics for 24 hours	Prophylactic antibiotics for five days	14 days	Infection rate at days 2, 5, and 14; effect of antibiotic duration on infection rate
Lawrentschuk et al., [15] (2002)	Australia	50	Hip surgery patients	PTG	NAA dressing	NR	Incidence of wound blisters adjacent to the surgical incision
Arti et al., [16] (2016)	Iran	90	Open fracture wounds/patients with open fractures	NPWT	Conventional wound dressing	1 month	Wound healing duration, incidence of infection, and hospitalization days

The included studies investigated a wide spectrum of surgical procedures. These included emergency laparotomy [9], endovascular aortic repair (EVAR) and open inguinal vascular surgery [10, 11], cesarean section [12], and orthopedic procedures such as open tibial fractures and hip surgery [13-16]. The primary intervention assessed was NPWT applied to closed incisions (iNPWT) in five of the trials [9-11, 13, 16]. Other advanced interventions studied were a DACC-impregnated dressing [12] and a paraffin-tulle gras (PTG) dressing [15]. The comparator in all trials was a form of conventional or standard surgical wound dressing. Follow-up durations for assessing the primary outcome of SSI incidence ranged from 14 days to 90 days postoperatively, with one trial including additional monitoring up to one year [9-12, 14].

Synthesis of Outcomes

The primary outcomes regarding SSI incidence and other relevant findings from the included RCTs are detailed in Table 2. The results demonstrate a variable effect of advanced wound therapies, particularly NPWT, across different surgical contexts.

**Table 2:** Summary of outcomes: effectiveness of NPWT and advanced interventions in preventing SSIs aOR: adjusted odds ratio; CI: confidence interval; DACC: dialkylcarbamoyl chloride; NAA: nonadherent absorbable; NPWT: negative pressure wound therapy; NR: not reported; OR: odds ratio; PTG: paraffin-tulle gras; RR: relative risk; SSD: standard surgical dressing; SSI: surgical site infection

Author (Year)	Intervention Group SSI Rate (%)	Control Group SSI Rate (%)	RR/OR
Atherton et al., [9] (2025)	28.4% (112/394)	27.4% (108/394)	RR = 1.03 (95% CI 0.83–1.28); P = 0.78
Svensson-Björk et al., [10] (2022)	Bilateral incisions: 1.8% (3/168)Unilateral incisions: 13.3% (2/15)	Bilateral incisions: 4.8% (8/168), Unilateral incisions: 11.5% (3/26)	Combined p = 0.49
Svensson-Björk et al., [11] (2021)	11.9% (7/59)	30.0% (18/60)	RR = 0.40 (approx.) / p = 0.015
Stanirowski et al., [12] (2016)	1.8% (DACC-impregnated dressing)	5.2% (SSD)	aOR = 2.94 for SSD vs DACC
Rezzadeh et al., [13] (2015)	NPWT (perioperative) – lowest complication rate	Wet-to-dry dressing-higher complication rate	NR
Ondari et al., [14] (2016)	23% (24-hour antibiotics)	19% (5-day antibiotics)	p = 0.699
Lawrentschuk et al., [15] (2002)	PTG: 8%	NAA: 64%	P = 0.0028
Arti et al., [16] (2016)	No significant difference, P=0.6	Conventional dressing: Not reported / low	NR

In major abdominal and vascular surgery, the evidence was mixed. The largest RCT, the SUNRRISE (Single-Use Negative-Pressure Dressing for Reduction in Surgical Site Infection Following Emergency Laparotomy) trial by Atherton et al. [9], found no significant benefit of iNPWT in preventing SSI after emergency laparotomy, with SSI rates of 28.4% in the iNPWT group and 27.4% in the control group (relative risk (RR) = 1.03, 95% CI 0.83-1.28; P = 0.78). Conversely, in vascular surgery, Svensson-Björk et al. [11] reported a substantially lower SSI rate with NPWT (11.9%) compared to standard dressings (30.0%) after open inguinal surgery, a difference that was statistically significant (p = 0.015). Their subsequent trial on EVAR patients [10] showed a non-significant trend towards lower SSI rates with NPWT in bilateral incisions (1.8% vs. 4.8%; combined p = 0.49).

In the context of cesarean section, Stanirowski et al. [12] demonstrated the effectiveness of a DACC-impregnated dressing, which was associated with a lower SSI rate (1.8%) compared to a standard surgical dressing (5.2%).

The outcomes in orthopedic and trauma patients were also heterogeneous. Rezzadeh et al. [13] reported that NPWT was associated with the lowest complication rate in severe open tibial fractures compared to wet-to-dry dressings, though specific statistical measures were not provided (NR). In contrast, Arti et al. [16] found no significant difference in infection incidence between NPWT and conventional dressings for open fracture wounds (P=0.6). A trial by Lawrentschuk et al. [15], which focused on wound blisters as a complication after hip surgery, found a significantly lower incidence with a PTG dressing (8%) compared to a nonadherent absorbable dressing (64%; P = 0.0028). Ondari et al. [14] investigated antibiotic duration rather than dressings and found no significant difference in infection rates between 24-hour and five-day prophylactic antibiotic regimens (p = 0.699).

Summary of Evidence

The synthesis of results from the eight RCTs indicates that the effectiveness of NPWT and other advanced wound interventions in preventing SSIs is not universal but is highly dependent on the surgical setting. A significant benefit was observed in specific patient populations, such as those undergoing open inguinal vascular surgery [11] and cesarean section [12]. However, in other common procedures like emergency laparotomy [9] and certain orthopedic trauma cases [16], advanced therapies like iNPWT did not demonstrate a statistically significant advantage over conventional dressings. Other outcomes, such as reduction in wound blisters [15] or overall complication rates in complex reconstructions [13], suggest that the benefits of these interventions may extend beyond SSI prevention alone in select scenarios.

Risk of Bias in Included Studies

The methodological quality of the eight included RCTs was assessed using the Cochrane RoB 2 tool, which revealed that the vast majority of studies were judged to have a low risk of bias. Specifically, the trials by Atherton et al. [9], Svensson-Björk et al. [10], Svensson-Björk et al. [11], Stanirowski et al. [12], Rezzadeh et al. [13], Ondari et al. [14], and Arti et al. [16] all demonstrated a low risk of bias across all five domains, including the randomization process, deviations from intended interventions, missing outcome data, measurement of the outcome, and selection of the reported result. In contrast, the trial by Lawrentschuk et al. [15] was rated with an overall high risk of bias due to some concerns in the domains of the randomization process, deviations from intended interventions, and selection of the reported result. Consequently, the body of evidence from the included RCTs is predominantly robust, with only one older study presenting notable methodological limitations (Table 3).

**Table 3:** Risk of bias assessment for included studies using the Cochrane Risk of Bias 2 (RoB 2) tool

Study (Author, Year)	D1: Randomization Process	D2: Deviations from Intended Interventions	D3: Missing Outcome Data	D4: Outcome Measurement	D5: Selection of Reported Result	Overall Risk of Bias
Atherton et al., [9] (2025)	Low	Low	Low	Low	Low	Low
Svensson-Björk et al., [10] (2022)	Low	Low	Low	Low	Low	Low
Svensson-Björk et al., [11] (2021)	Low	Low	Low	Low	Low	Low
Stanirowski et al., [12] (2016)	Low	Low	Low	Low	Low	Low
Rezzadeh et al., [13] (2015)	Low	Low	Low	Low	Low	Low
Ondari et al., [14] (2016)	Low	Low	Low	Low	Low	Low
Lawrentschuk et al., [15] (2002)	Some concerns	Some concerns	Low	Low	Some concerns	High
Arti et al., [16] (2016)	Low	Low	Low	Low	Low	Low

Discussion

This systematic review evaluated the effectiveness of NPWT and other advanced interventions in preventing SSIs by synthesizing evidence from eight RCTs across diverse surgical settings. The overarching finding is that the efficacy of these advanced wound management strategies is not uniform but is profoundly contingent upon the specific surgical context, patient population, and the comparator being used. The evidence does not support the universal application of advanced dressings like iNPWT for all surgical procedures; instead, it paints a nuanced picture where significant benefits are observed in specific, high-risk scenarios, while in other common procedures, they offer no discernible advantage over standard care. This contextual dependency is critical for clinicians, healthcare policymakers, and researchers to understand, as it moves the conversation beyond a simple "is it effective?" to the more pertinent question of "for whom, and under what conditions, is it effective?"

The most striking finding of this review is the clear divergence in outcomes between different surgical specialties. In the domain of open inguinal vascular surgery, the evidence from Svensson-Björk et al. [11] is compelling, demonstrating a substantial and statistically significant reduction in SSI rates with iNPWT (11.9%) compared to standard dressings (30.0%). This aligns with the pathophysiological rationale that groin incisions in vascular patients are particularly susceptible to complications due to factors like lymphatic leakage, proximity to the perineum, and often compromised vascularity. The closed, sealed environment of iNPWT may mitigate these risks by effectively managing exudate, stabilizing the incision, and enhancing perfusion. This finding is supported by a meta-analysis by Hyldig et al. [17] focusing on closed incisions, which concluded that iNPWT significantly reduced SSIs, particularly in orthopedic and cardiothoracic procedures, suggesting a shared benefit in incisions over high-motion areas or in patients with comorbidities. Similarly, a systematic review by O'Leary et al. [18] found a consistent, though not always significant, trend towards SSI reduction with iNPWT in a variety of settings, highlighting its potential in targeted applications.

Conversely, the results from the large, multi-center SUNRRISE trial by Atherton et al. [9] in emergency laparotomy patients are a powerful counterpoint. The finding of virtually identical SSI rates between the iNPWT and standard dressing groups (28.4% vs. 27.4%) challenges the assumption that iNPWT is a panacea for SSI prevention in major abdominal surgery. This suggests that the risk factors for SSI in a contaminated or dirty emergency laparotomy, such as intra-abdominal soilage, patient frailty, and systemic inflammatory response, may be so dominant that they cannot be overcome by a localized intervention at the incision level alone. This outcome resonates with the findings of the 2018 RCT by Sahebally et al. [19], which also found no significant difference in SSI rates between iNPWT and standard dressings in elective colorectal surgery, indicating that the abdominal wall itself may not derive the same benefit from iNPWT as groin incisions. The high baseline SSI rate in both groups of the SUNRRISE trial underscores the multifactorial nature of SSI in this population and implies that broader, system-wide bundles of care might be more impactful than a focus on the dressing alone.

In obstetric surgery, the trial by Stanirowski et al. [12] demonstrated the effectiveness of a DACC-impregnated dressing, which reduced SSI rates from 5.2% to 1.8% following cesarean section. This is a significant finding, as cesarean sections are among the most common surgical procedures globally. The mechanism of DACC dressings, which involves binding and inactivating microorganisms, appears to offer a tangible benefit in this clean-contaminated procedure. This result finds support in the work of Lavigne et al. [20], who, in a study on sternal wounds, found that a DACC-coated dressing was associated with a lower incidence of SSI compared to standard dressings, reinforcing the potential of antimicrobial-impregnated dressings as a valuable tool in the SSI prevention arsenal, particularly where the primary bacterial burden is from skin flora.

The evidence from orthopedic and trauma surgery presented in this review is notably heterogeneous, reflecting the diversity of injuries and surgical approaches within this field. The study by Rezzadeh et al. [13] suggested that NPWT was associated with the lowest complication rate in severe open tibial fractures (Gustilo IIIB/C), a population at extreme risk for infection and non-union. This aligns with the well-established role of NPWT in the management of open wounds, where it aids in edema reduction, tissue granulation, and wound bed preparation. This is consistent with the guidelines from the International Consensus Meeting on Musculoskeletal Infection, which recommends NPWT for the temporary management of open fractures prior to definitive closure. However, the study by Arti et al. [16] found no significant difference in infection rates between NPWT and conventional dressings for open fracture wounds. This discrepancy may be explained by differences in fracture severity, the timing and duration of NPWT application, or the specific protocols for wound debridement and antibiotic therapy. The negative finding from Arti et al. [16] echoes a certain caution found in the literature; for instance, a meta-analysis by Liu et al. [21] concluded that while NPWT may reduce the risk of infection in open fractures, the evidence quality was low, and more robust RCTs are needed. The significant result from Lawrentschuk et al. [15], showing a dramatic reduction in wound blisters with a PTG dressing after hip surgery, highlights an important, though often overlooked, aspect of wound care. While blisters are not infections, they represent a failure of the skin barrier and can be a precursor to more serious complications, including SSI. This finding is supported by a study by Naylor et al. [22], which also identified specific dressing types as a modifiable risk factor for blister formation, suggesting that simple, cost-effective advanced dressings can improve patient comfort and potentially prevent downstream issues.

It is also crucial to address the trial by Ondari et al. [14], which, while not testing a dressing, investigated antibiotic duration in open tibia fractures and found no difference between 24-hour and five-day regimens. This serves as a critical reminder that advanced technologies like NPWT are not a substitute for fundamental, evidence-based practices like appropriate antibiotic prophylaxis. The finding aligns with a growing body of literature, such as the systematic review by Slobogean et al. [23], which supports short-course antibiotic prophylaxis in open fractures, suggesting that resources might be better allocated to ensuring adherence to such foundational principles rather than universally adopting costly advanced dressings.

When interpreting these collective findings, the assessment of the risk of bias is paramount. The conclusion that the majority of the included trials had a low risk of bias [9-14, 16] lends considerable weight to the synthesized results, particularly the null findings of the large SUNRRISE trial [9] and the positive findings in vascular surgery [11]. The robustness of these methodologically sound studies strengthens the argument for context-specific application. The high risk of bias identified in the older trial by Lawrentschuk et al. [15] necessitates that its compelling findings be viewed with caution, though they remain hypothesis-generating and point to an area worthy of further investigation with more rigorous modern RCTs.

The synthesis of this evidence compels a re-evaluation of the role of advanced wound therapies. The enthusiasm for iNPWT, driven by compelling physiological theories and early positive studies, must be tempered by the reality of high-quality, contemporary evidence. The concept of "value-based healthcare" becomes central here. For a high-risk patient population like those undergoing open vascular surgery, where the cost of an SSI is immense in terms of patient morbidity, graft loss, and financial burden, the investment in iNPWT appears justified based on the evidence from Svensson-Björk et al. [11]. In contrast, for emergency laparotomy, where the SUNRRISE trial [9] found no benefit, the routine use of iNPWT would represent a substantial and unjustifiable cost without proven clinical benefit. This echoes the sentiments of a health technology assessment by Shiroky et al. [24], which concluded that the cost-effectiveness of iNPWT is highly sensitive to the baseline SSI rate and the absolute risk reduction achieved; it is likely cost-saving only in populations with very high baseline risk. Therefore, the future of these interventions lies not in blanket adoption, but in the strategic identification of patient and procedure-specific risk factors that predict a high likelihood of benefit. Future research should focus on developing and validating risk prediction models to guide the cost-effective deployment of iNPWT and other advanced dressings, moving towards a more personalized approach to surgical wound management.

Limitations

This systematic review has several limitations. Firstly, the number of included studies was relatively small (n=8), and they exhibited significant clinical heterogeneity in terms of surgical procedures, patient populations, and specific interventions, precluding a meaningful meta-analysis and statistical pooling of the results. Secondly, the definition of the "control" dressing varied across studies, ranging from simple gauze to more modern adherent dressings, which could influence the magnitude of the observed effect. Thirdly, the follow-up durations differed, and some SSIs, particularly those that are deep or organ-space, may present outside the standard 30-day window, potentially leading to an underestimation of the true infection rate in some trials. Finally, while the overall risk of bias was low for most studies, the inherent challenge of blinding in trials involving physical dressings introduces a potential for performance bias.

## Conclusions

This systematic review synthesized evidence from eight RCTs that were too clinically heterogeneous, encompassing diverse surgical populations, interventions, and comparators, to permit a quantitative meta-analysis. Consequently, a narrative synthesis was performed. The findings consistently demonstrate that the effectiveness of NPWT and advanced wound interventions in preventing SSIs is not universal but is profoundly specific to the surgical context. Significant benefits were observed in targeted scenarios such as open inguinal vascular surgery and cesarean section with DACC dressings, while no advantage was found in emergency laparotomy. Therefore, the evidence supports a selective, rather than a routine, application of these technologies. Clinical implementation should be guided by procedure-specific risk assessments and a consideration of cost-effectiveness, focusing on patient populations most likely to derive a meaningful benefit. Future research should prioritize the identification of robust predictive factors for SSI in different surgical contexts to enable the precise and personalized use of advanced wound care therapies.

## Appendices

Appendix A 

**Table 4:** Search strategies for the databases

Database	Search String / Keywords	Notes / Filters
ClinicalTrials.gov	(“Negative Pressure Wound Therapy” OR NPWT OR “advanced wound care” OR “advanced intervention”) AND (“surgical site infection” OR SSI OR “postoperative infection”)	Use filters: “Recruiting, Active, Completed”; Study type: Interventional / Observational; Time frame: All years
PubMed	(“Negative Pressure Wound Therapy”[Title/Abstract] OR NPWT[Title/Abstract] OR “advanced wound care”[Title/Abstract]) AND (“surgical site infection”[Title/Abstract] OR SSI[Title/Abstract] OR “postoperative infection”[Title/Abstract])	Filters: Humans, English, Last 10 years; Include RCTs, Clinical Trials, Systematic Reviews
Embase (Elsevier)	(‘negative pressure wound therapy’ OR NPWT OR ‘advanced wound care’ OR ‘advanced intervention’) AND (‘surgical site infection’ OR SSI OR ‘postoperative infection’)	Filters: Humans, English, Articles, Conference abstracts excluded; Use Emtree terms if needed
Scopus	TITLE-ABS-KEY(“negative pressure wound therapy” OR NPWT OR “advanced wound care” OR “advanced intervention”) AND TITLE-ABS-KEY(“surgical site infection” OR SSI OR “postoperative infection”)	Filters: English, Document type: Article / Review; All years
Web of Science	TS=(“negative pressure wound therapy” OR NPWT OR “advanced wound care” OR “advanced intervention”) AND TS=(“surgical site infection” OR SSI OR “postoperative infection”)	Filters: English, Document types: Article / Review; Timespan: All years
